# Weight-adjusted-waist index is positively associated with urinary incontinence: results from the National Health and Nutrition Examination Survey (NHANES) 2001–2018

**DOI:** 10.1186/s40001-024-01971-9

**Published:** 2024-07-16

**Authors:** Shangqi Cao, Xu Hu, Yaxiong Tang, Kang Wu, Weixiao Yang, Xiang Li

**Affiliations:** Department of Urology, Institute of Urology, West China Hospital, Sichuan University, 37 Guoxue Alley, Chengdu, 610041 China

**Keywords:** Weight-adjusted-waist index, Urinary incontinence, National Health and Nutrition Examination Survey, NHANES, Obesity

## Abstract

**Background:**

Urinary incontinence (UI) is closely related to obesity. The aim of this study is to evaluate the association of a novel anthropometric indicator weight-adjusted-waist index (WWI) with UI.

**Methods:**

This cross-sectional study used the data from National Health and Nutrition Examination Survey (NHANES) 2001–2018. Weighted multivariable logistic regression was used to evaluate the relationship between WWI and three types of UI [stress UI (SUI), urgency UI (UUI), and mixed UI (MUI)]. The receiver operating characteristic (ROC) curve and Delong et al.’s test were utilized for comparison of the predictive capability for UI between WWI and body mass index (BMI), waist circumference (WC).

**Results:**

A total of 41,614 participants were included in this study, of whom 23.57% had SUI, 19.24% had UUI, and 9.43% had MUI. In the fully adjusted model, WWI was positively associated with three types of UI [SUI: odds ratio (OR) = 1.19, 95%Confidence interval (CI) 1.13–1.25; UUI: OR = 1.18, 95%CI 1.13–1.24; MUI: OR = 1.19, 95%CI 1.11–1.27, all *p* < 0.001]. Compared to the lowest WWI interval, the positive correlation between WWI and UI still existed in the highest WWI group after converting WWI to a categorical variable by quartiles (SUI: OR = 1.52, 95%CI 1.35–1.71, *p* < 0.001; UUI: OR = 1.50, 95%CI 1.33–1.69, *p* < 0.001; MUI: OR = 1.55, 95%CI 1.32–1.83, *p* < 0.001). WWI had a stronger prediction for three types of UI than BMI and WC (all *p* < 0.001).

**Conclusion:**

A higher WWI was linked with an increased likelihood of three types of UI (SUI, UUI, and MUI) in the United State population. Compared to BMI and WC, WWI had a stronger predictive power for UI. WWI may be a better adiposity parameter for evaluating UI.

## Introduction

Urinary incontinence (UI) is a common disease worldwide and is defined as the involuntary loss of urine [1]. On a global scale, the prevalence of UI exhibited a positive connection with advancing age, whereby up to 30% to 40% of elderly women acknowledged experiencing UI [2]. A National Health and Nutrition Examination Survey (NHANES) cross-sectional study indicated that 17.1% of American women aged 20 years or older complaint of moderate-to-severe UI [3]. It demonstrated that UI had a documented prevalence of 11% among men aged 60–64 years, escalating to 31% in men aged 85 years and above [4]. UI is mainly classified as stress urinary incontinence (SUI), urgency urinary incontinence (UUI), and mixed urinary incontinence (MUI) [5]. Based on the International Continence Society, SUI is defined as involuntary urine leakage during coughing, sneezing, or physical exertion. UUI is described as involuntary urine loss associated with urgency. MUI is expressed as the complaint of involuntary loss of urine associated with urgency and with effort, exertion, coughing, or sneezing [6]. UI is a common health problem that affects the daily life of many people and imposes a huge economic burden on society [7]. Numerous risk factors contribute to UI, including but not limited to aging, childbirth complications, medications, sedentary behavior, and obesity [2].

Obesity is a severe public health threat with a significantly increased prevalence worldwide [8]. Ward et al. predicted that almost half of American adults will have obesity by 2030 [9]. Some traditional adiposity indicators such as body mass index (BMI) and waist circumference (WC) are widely used to evaluate the degree of obesity. However, there are some limitations in the assessment of obesity using these common parameters. For instance, the inability of BMI to distinguish body fat and lean mass resulted in limited diagnostic accuracy in individuals with intermediate BMI ranges [10]. WC is considered a simple and convenient indicator for evaluating abdominal or central obesity and reflect the visceral adiposity tissue [11]. However, the strong correlation between WC and BMI resulted in that WC was not free from the impact of BMI [12]. Weight-adjusted-waist index (WWI) was proposed by Park et al. [13] as a novel adiposity indicator and calculated as WC (cm)/weight (kg)^1/2^. WWI had a good predictive performance for cardiometabolic disorders, cardiovascular mortality, and all-cause mortality [13]. Moreover, Kim et al. indicated that WWI was positively related to fat mass whereas negatively associated with muscle mass in participants older than 65 years old [14].

Epidemiological surveys demonstrated that obesity is regarded as an independent risk factor for the prevalence of UI. A meta-epidemiology study indicated that middle-aged and older women with overweight (25 kg/m^2^ ≤ BMI < 30 kg/m^2^) and obesity (30 kg/m^2^ ≤ BMI < 35 kg/m^2^) had a higher risk of UI [15]. Park et al. showed that visceral obesity may be stronger associated with UI compared with overall obesity [16]. Choi et al. demonstrated that there was an association between a chronic increase in BMI and a higher risk of UI in the later stages of life. In addition, the increased duration of being either overweight or obese resulted in more severe symptoms of UI [17]. However, to our knowledge, the association between WWI and the risk of UI has not been investigated. Therefore, it is necessary to explore the relationship between WWI and three types of UI (SUI, UUI, and MUI) using the data obtained from the NHANES database ranging from 2001 to 2018.

## Materials and methods

### Study description and population

The analyzed data in this cross-sectional study were collected from NHANES, a population-based survey conducted by the Centers for Disease Control and Prevention’s National Center for Health Statistics (NCHS). NHANES is designed to assess the health and nutritional status of the United States population. The integration of in-home interviews and physical examinations represents a distinctive characteristic of the survey methodology. The component of in-home interview encompasses demographics, socioeconomic status, dietary habits, and health-related information. The physical examinations comprise medical, dental, and physiological assessments, as well as laboratory tests, all administered by qualified medical professionals. The NHANES used a complex stratified multistage probability design to obtain a representative sample of the resident civilian non-institutionalized U.S. population [18]. More detailed information can be accessed at https://www.cdc.gov/nchs/nhanes/index.htm.

We enrolled survey individuals who completed the examination of body measures and the questionnaire on kidney conditions from nine NHANES cycles 2001–2018 at first, since the examination of body measures provided the WC and body weight data for the calculation of WWI and the questionnaire on kidney conditions included the interview for the evaluation of UI. The participant exclusion criteria were as follows: (1) participants without complete WC and weight data; (2) participants without answering the interview about assessing the condition of UI; (3) participants with missing data on other potential covariates.

### Measurement of WWI

In this study, WWI was considered an exposure variable calculated as WC (cm) divided by the square root of weight (kg) [13]. The information on WC and weight was available in the section “Body Measures” of the NHANES examination data. As a novel adiposity index, an elevated WWI reveals a more severe degree of obesity. WWI was designed as continuous and categorical variables in analyses. The categorical WWI was divided into four subgroups (Q1–Q4) based on the WWI quartiles.

### Assessment of UI

There were two questions evaluating the conditions of UI in NHANES. If participants had answered yes to the question “During the past 12 months, have you leaked or lost control of even a small amount of urine with an activity like coughing, lifting or exercise?”, they were defined as stress UI (SUI). If survey individuals had responded yes to the question “During the past 12 months, have you leaked or lost control of even a small amount of urine with an urge or pressure to urinate and you couldn't get to the toilet fast enough?”, they were defined as urgency UI (UUI). Participants who answered yes to both the above questions were considered mixed UI (MUI).

### Covariates of interest

In this study, gender, age, race/ethnicity, education level, marital status, the family poverty income ratio (PIR), BMI, physical activity (vigorous/moderate), smoking status, alcohol intaking, diabetes, and hypertension were set as the covariates of interest. Numerous missing covariates for the family PIR (*n* = 3265) and alcohol intaking (*n* = 604) were designed as missing value categories to avoid further reducing huge samples in our study, and the missing value categories were designed as dummy variables in regression models. If participants engaged in any vigorous activities including running, lap swimming, aerobics classes, or brisk bicycling for a duration of at least 10 min during the past 30 days that resulted in heavy sweating, or notable increases in breathing or heart rate, they were set as having vigorous activities. Participants who did moderate activities such as brisk walking, leisurely bicycling, golf, and dancing for a minimum of 10 min, resulting in only light sweating or a slight to moderate elevation in breathing or heart rate during the past 30 days were designed as having moderate activities. If survey individuals had smoked at least 100 cigarettes throughout their entire life and smoked every day or some days at the time of the questionnaire, they were defined as current smokers. Participants who smoked at least 100 cigarettes in their entire life and did not smoke when taking the questionnaire were set as former smokers. Additionally, if individuals answered that they smoked less than 100 cigarettes during their lifetime, they were considered nonsmokers. Participants were categorized as drinkers and nondrinkers (whether had at least 12 alcohol drinks per year). If participants were diagnosed with diabetes by doctors before the interview or their fasting plasma glucose was ≥ 126 mg/dL, they were regarded as having diabetes. If doctors told survey individuals that they had hypertension, or participants were taking a prescription for hypertension, or their systolic blood pressure was ≥ 140 mmHg, or their diastolic blood pressure was ≥ 90 mmHg, they were considered having hypertension.

### Statistical analysis

In the current study, considering the NHANES complex multistage sampling design, appropriate sampling weights, stratification, and clustering were utilized in all statistical analyses. Continuous variables were presented as weighted mean and standard error (SE), and categorical variables were expressed as weighted proportions. To compare the differences among four groups divided by WWI quartiles, a survey-weighted linear regression for continuous variables and a survey-weighted Chi-square test for categorical variables were used in the baseline characteristics table.

The association between WWI and UI was accessed by multivariable logistic regressions in three different models. Model 1, no covariates were adjusted; Model 2 was adjusted for gender, age, and race; Model 3 was adjusted for gender age, race, BMI, education level, marital status, the family PIR, smoking status, alcohol intaking, vigorous activity, moderate activity, diabetes, and hypertension. In subgroup analysis, stratified multivariable logistic regression models were utilized to explore the relationship between WWI and UI in different subgroups. The predictive capability of WWI, BMI and WC for UI was evaluated by the receiver operating characteristic (ROC) curve and the area under curve (AUC). Delong et al.’s test [19] was used to compare the difference in AUC between WWI and BMI, WC. A two-sided *p* value of < 0.05 was considered statistically significant. All statistical analyses were conducted using EmpowerStats (http://www.empowerstats.com, X&Y Solutions, Inc.) and statistical software packages R (http://www.R-project.org; The R Foundation).

## Results

**Fig. 1:**
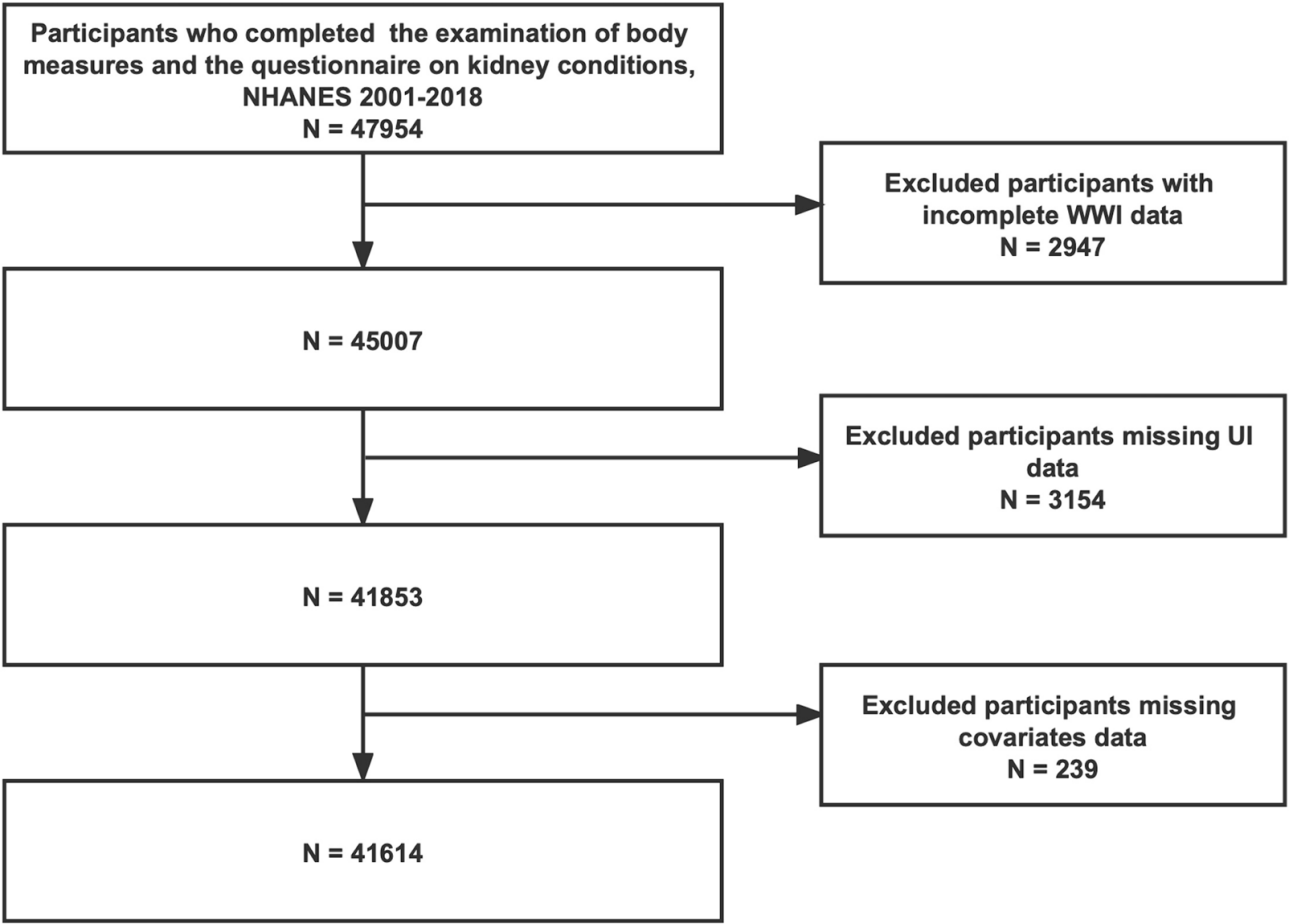
Flow chart of sample selection

A total of 47,954 participants who completed the examination of body measures and the questionnaire on kidney conditions were enrolled at first. We excluded participants with missing WWI data (*n* = 2947), incomplete UI data (*n* = 3154), missing covariates data [missing BMI data (*n* = 123), education level (*n* = 33), marital status (*n* = 17), hypertension (*n* = 12), diabetes (*n* = 13), vigorous activity (*n* = 7), moderate activity (*n* = 10), and smoking status (*n* = 24)]. Finally, 41,614 survey individuals were included in this study (Fig. 1).

### Participant characteristics

The weighted baseline characteristics are shown in Table 1. A total of 41,614 participants (48.81% males and 51.19% females, weighted proportions) with a weighted mean (SE) age of 46.98 (0.20) years were enrolled in this study. Of these participants, 23.57% had a self-reported history of SUI, 19.24% complained of UUI, and 9.43% had MUI. WWI was set as four categories (7.59–10.47, 10.48–11.05, 11.06–11.63, and 11.64–15.70) by the quartiles. Participants with an elevated WWI quartile had a higher prevalence of all types of UI (all *p* < 0.001).

**Table 1:** Baseline characteristics of participants by the WWI quartiles

	Total	Q1 (7.59–10.47)	Q2 (10.48–11.05)	Q3 (11.06–11.63)	Q4 (11.64–15.70)	*p* value
Participants (*n*)	41,614	10,404	10,403	10,402	10,405	
Age (year), mean (SE)	46.98 (0.20)	37.27 (0.23)	45.61 (0.21)	51.74 (0.25)	57.34 (0.26)	< 0.001
BMI (kg/m^2^), mean (SE)	28.82 (0.07)	24.81 (0.07)	28.08 (0.07)	30.44 (0.09)	33.73 (0.13)	< 0.001
Gender (%)						< 0.001
Male	48.81	56.14	53.22	47.69	33.72	
Female	51.19	43.86	46.78	52.31	66.28	
Age (%)						< 0.001
< 50	56.67	80.95	61.15	43.71	30.65	
≥ 50	43.33	19.05	38.85	56.29	69.35	
Race/ethnicity (%)						< 0.001
Mexican American	8.15	5.13	8.53	10.05	9.82	
Other Hispanic	5.25	4.53	5.51	5.51	5.64	
Non-Hispanic White	69.03	69.09	68.67	68.35	70.19	
Non-Hispanic Black	10.84	14.30	9.69	9.60	8.80	
Other race	6.73	6.95	7.60	6.48	5.56	
Education level (%)						< 0.001
Less than high school	15.93	10.90	13.85	18.65	22.80	
High school or GED	23.95	20.39	23.43	25.86	27.58	
Above high school	60.12	68.72	62.72	55.49	49.63	
Marital status (%)						< 0.001
Living alone	35.82	40.90	31.05	30.99	40.35	
Married or living with partner	64.18	59.10	68.95	69.01	59.65	
Family PIR (%)						< 0.001
≤ 1.3	19.19	17.15	16.89	19.66	24.65	
> 1.3 and ≤ 3.5	33.68	31.38	32.55	34.19	37.87	
> 3.5	40.75	45.79	44.51	39.83	29.59	
Unclear	6.38	5.67	6.05	6.31	7.89	
BMI (%)						< 0.001
< 25	30.66	58.11	28.72	16.99	9.35	
≥ 25 and < 30	33.35	30.36	40.39	36.10	25.20	
≥ 30	35.99	11.53	30.88	46.91	65.45	
Hypertension (%)						< 0.001
No	62.85	82.39	66.59	54.18	39.72	
Yes	37.15	17.61	33.41	45.82	60.28	
Diabetes (%)						< 0.001
No	89.44	97.63	93.57	87.15	74.80	
Yes	10.56	2.37	6.43	12.85	25.20	
Vigorous activity (%)						< 0.001
No	73.70	63.83	72.21	78.18	84.74	
Yes	26.30	36.17	27.79	21.82	15.26	
Moderate activity (%)						< 0.001
No	52.85	46.63	51.40	54.98	61.29	
Yes	47.15	53.37	48.60	45.02	38.71	
Smoking status (%)						< 0.001
Current smokers	21.23	23.39	22.29	19.98	18.16	
Former smokers	25.00	17.60	23.97	29.85	31.46	
Nonsmokers	53.77	59.01	53.75	50.17	50.38	
Alcohol intaking (%)						< 0.001
Nondrinkers	26.19	19.45	23.10	28.26	37.61	
Drinkers	72.77	79.82	75.94	70.76	60.74	
Unclear	1.04	0.73	0.96	0.98	1.65	
SUI (%)						< 0.001
No	76.43	85.82	79.24	73.18	62.90	
Yes	23.57	14.18	20.76	26.82	37.10	
UUI (%)						< 0.001
No	80.76	89.59	83.74	77.68	67.64	
Yes	19.24	10.41	16.26	22.32	32.36	
MUI (%)						< 0.001
No	90.57	95.84	92.72	88.85	82.08	
Yes	9.43	4.16	7.28	11.15	17.92	

*Q1–Q4* quartile 1-quartile 4, *SE* standard error, *WWI* weight-adjusted-waist index, *BMI* body mass index, *GED* general educational development, *Family PIR* family poverty income ratio, *SUI* stressed urinary incontinence, *UUI* urgency urinary incontinence, *MUI* mixed urinary incontinence

### Association between WWI and UI

Weighted multivariable logistic regression models were used to evaluate the relationship between WWI and UI in crude, minimally, and fully adjusted models (Model 1, Model 2, and Model 3, respectively), and WWI was designed as a continuous and categorical variable (Q1–Q4) in the analysis. In model 3, a one-unit increase in WWI was related to the higher odds of all types of UI [SUI: odds ratio (OR) = 1.19, 95%Confidence interval (95%CI) 1.13–1.25; UUI: OR = 1.18, 95%CI 1.13–1.24; MUI: OR = 1.19, 95%CI 1.11–1.27, all *p* < 0.001, Table 2]. In addition, the further analysis indicated that survey individuals in the highest WWI quartile (Q4) had increased risks of all types of UI than those in the lowest WWI quartile (Q1) in model 3 (SUI: OR = 1.52, 95%CI 1.35–1.71; UUI: OR = 1.50, 95%CI 1.33–1.69; MUI: OR = 1.55, 95%CI 1.32–1.83, all *p* for trend < 0.001, Table 2).

**Table 2:** Association between weight-adjusted-waist index with urinary incontinence

SUI	OR (95%CI), *p* value		
	Model 1	Model 2	Model 3
Continuous	1.81 (1.75, 1.88), < 0.001	1.40 (1.34, 1.46), < 0.001	1.19 (1.13, 1.25), < 0.001
Categories			
Q1	Reference	Reference	Reference
Q2	1.59 (1.45, 1.74), < 0.001	1.49 (1.35, 1.66), < 0.001	1.28 (1.15, 1.42), < 0.001
Q3	2.22 (2.02, 2.44), < 0.001	1.84 (1.66, 2.04), < 0.001	1.41 (1.27, 1.57), < 0.001
Q4	3.57 (3.27, 3.90), < 0.001	2.18 (1.96, 2.43), < 0.001	1.52 (1.35, 1.71), < 0.001
*p* for trend	< 0.001	< 0.001	< 0.001

UUI	OR (95%CI), *p *value		
	Model 1	Model 2	Model 3
Continuous	1.90 (1.83, 1.98), < 0.001	1.41 (1.36, 1.48), < 0.001	1.18 (1.13, 1.24), < 0.001
Categories			
Q1	Reference	Reference	Reference
Q2	1.67 (1.49, 1.87), < 0.001	1.36 (1.21, 1.53), < 0.001	1.20 (1.07, 1.35), 0.003
Q3	2.47 (2.23, 2.75), < 0.001	1.64 (1.47, 1.83), < 0.001	1.31 (1.16, 1.47), < 0.001
Q4	4.12 (3.74, 4.53), < 0.001	2.20 (1.98, 2.45), < 0.001	1.50 (1.33, 1.69), < 0.001
*p* for trend	< 0.001	< 0.001	< 0.001

MUI	OR (95%CI), *P*-value		
	Model 1	Model 2	Model 3
Continuous	2.08 (1.97, 2.19), < 0.001	1.50 (1.41, 1.58), < 0.001	1.19 (1.11, 1.27), < 0.001
Categories			
Q1	Reference	Reference	Reference
Q2	1.81 (1.55, 2.11), < 0.001	1.51 (1.29, 1.77), < 0.001	1.27 (1.08, 1.49), 0.005
Q3	2.89 (2.52, 3.32), < 0.001	1.99 (1.73, 2.29), < 0.001	1.46 (1.26, 1.69), < 0.001
Q4	5.04 (4.37, 5.80), < 0.001	2.59 (2.22, 3.02), < 0.001	1.55 (1.32, 1.83), < 0.001
*p* for trend	< 0.001	< 0.001	< 0.001

*OR* odds ratio, *95%CI* 95%Confidence interval

Model 1: unadjusted; Model 2: adjusted for gender, age, and race/ethnicity; Model 3: adjusted for gender, age, race/ethnicity, body mass index, education level, marital status, the family poverty income ratio, smoking status, alcohol intaking, vigorous activity, moderate activity, diabetes, and hypertension

### Subgroup analysis

**Fig. 2:**
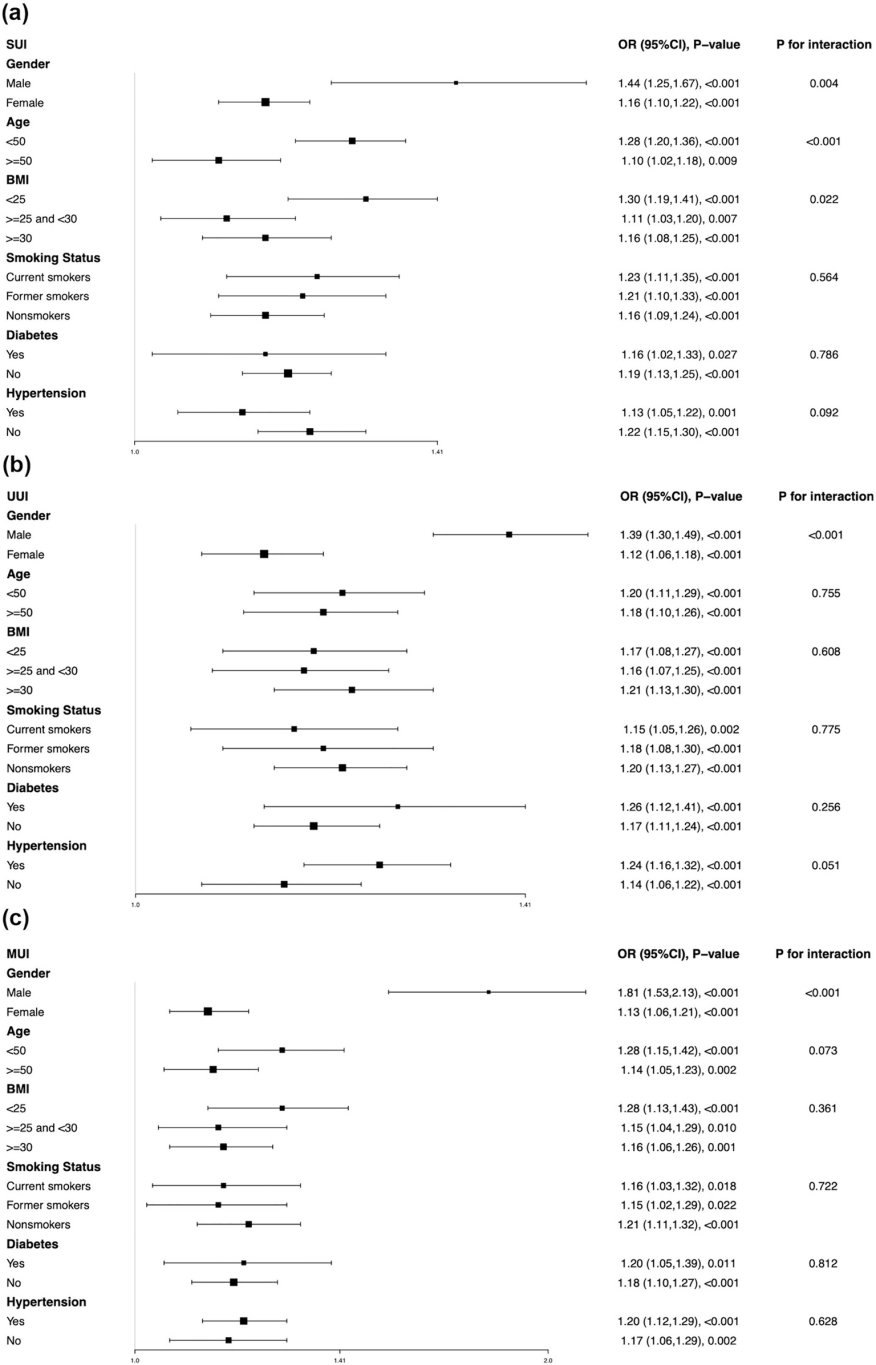
Subgroup analysis for the correlation between WWI and three types of UI. Stratified factors included gender, age, BMI, smoking status, diabetes, and hypertension. All analyses were adjusted by gender, age, race, education, marital status, the family PIR, BMI, hypertension, diabetes, vigorous activity, moderate activity, smoking status, and alcohol intaking, except the stratified factor itself

Subgroup analysis was conducted to explore the potential factors affecting the relationship between WWI and UI. As shown in Fig. 2a, gender, age, and BMI potentially affect the association between WWI and SUI with full adjustment of all covariates except the stratified factor itself (all *p* for interaction < 0.05). Additionally, in stratified multivariable logistic regression for UUI and MUI (Fig. 2b, c, respectively), stronger relationships were observed in male participants than in females (both *p* for interaction < 0.001). Furthermore, WWI was still significantly positively correlated with the prevalence of three types of UI in all analyzed subgroups. Detailed information has been shown in Fig. 2.

### The predictive performance of WWI, BMI, and WC for UI

**Fig. 3:**
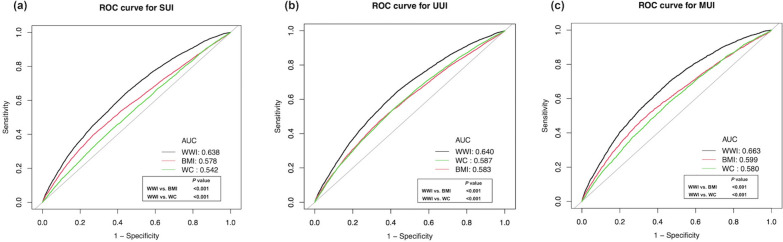
Receiver operating characteristic (ROC) curve analysis and Delong et al.’s test for comparison of the predictive power between WWI and BMI, WC for three types of UI

The AUC values of three adiposity indicators for predicting three types of UI were shown: 0.638 vs. 0.578 vs. 0.542 (WWI vs. BMI vs. WC for SUI); 0.640 vs. 0.583 vs. 0.587 (WWI vs. BMI vs. WC for UUI); 0.663 vs. 0.599 vs. 0.580 (WWI vs. BMI vs. WC for MUI). WWI had the highest AUC value for predicting all types of UI among the three anthropometric indexes (Table 3). The ROC curve and Delong et al.’s test compared the differences in AUC values for predicting all types of UI between WWI and BMI, WC and found that WWI had a better predictive ability for UI than BMI and WC (all *p* < 0.001) (Fig. 3).

**Table 3:** The adiposity indicators for predicting UI

	AUC	95%CI low	95%CI upp	Cutoff value	Specificity	Sensitivity
SUI						
WWI	0.638	0.632	0.644	11.076	0.560	0.640
BMI	0.578	0.571	0.584	31.185	0.724	0.403
WC	0.542	0.535	0.549	101.65	0.609	0.452
UUI						
WWI	0.640	0.638	0.646	11.082	0.563	0.649
BMI	0.583	0.577	0.590	29.885	0.654	0.478
WC	0.587	0.580	0.593	100.050	0.584	0.545
MUI						
WWI	0.663	0.655	0.671	11.080	0.540	0.698
BMI	0.599	0.590	0.609	30.785	0.690	0.470
WC	0.580	0.571	0.589	98.950	0.539	0.580

*AUC* area under curve, *95%CI* 95%Confidence interval

## Discussion

In the current study, we used the data from the NHANES database 2001–2018 to investigate the relationship between WWI and UI in the United State non-institutionalized residents. The results demonstrated that a higher WWI was associated with a greater risk of three types of UI. Additionally, regarding WWI as a categorical variable, the strong positive association between WWI and the prevalence of three types of UI was still observed. Furthermore, subgroup analysis indicated that gender, age, and BMI were potential factors for the association between WWI and SUI, and gender was the effect modifier for the relationship between WWI and UUI, MUI. Moreover, in all subgroups, WWI was significantly positively linked with the increased likelihood of UI. Lastly, the ROC curve and Delong et al.’s test were used to evaluate the predictive power of WWI, BMI, and WC for UI, and found that WWI had a better predictive performance for three types of UI.

It has been shown that some factors are closely associated with the incidence of UI, including but not limited to age, parity, obesity, diabetes mellitus, the history of hysterectomy or pelvic surgery, and cardiorespiratory diseases [20]. While obesity has been confirmed as a recognized risk factor of UI, the connection between obesity and UI is not clear. It is speculated that elevated body weight increases abdominal pressure, and after that bladder pressure and urethral mobility increase, which results in SUI [21]. Additionally, obesity increases abdominal pressure, consequently exacerbating detrusor instability, leading to UUI [22]. Many studies investigated the positive association between obesity and UI. The Finnish National Nocturia and Overactive Bladder Study showed that obesity was connected with a higher risk of SUI (OR = 1.9, 95%CI 1.2–3.0) and UUI (OR = 3.0, 95%CI 1.2–7.4) in women [23]. An elderly EXERNET multi-center study found that compared to women without UI, higher BMI, body fat percentage, and WC were observed in postmenopausal females elder than 65 years old with UI (all *p* < 0.05) [24]. According to a Korean National Health and Nutrition Examination Survey, Park et al. used dual energy X-ray absorptiometry (DEXA) to evaluate the association between obesity and UI and discovered that many adiposity indexes were positively correlated with UI in women [25]. In addition, central obesity is closely associated with UI. Han et al. demonstrated that a positive relationship between SUI and abdominal obesity was observed in Korean women [26]. A cross-sectional survey including 19,024 women in China showed that central obesity (WC ≥ 80 cm of women) was considered a potential risk factor for SUI [27]. Furthermore, loss of muscle mass was also related to UI. Erdogan et al. indicated that UI was independently related to sarcopenia when muscle mass was adjusted by weight and to low muscle mass when muscle mass was adjusted by weight or BMI [28]. A prospective observational cohort study found that women aged 70 or older had higher odds of new or persistent SUI if their muscle grip strength decreased by 5% or more (adjusted OR = 1.60, *p* = 0.04) [29]. Parker-Autry et al. demonstrated that compared to women without incident UI, women with incident UI had greater odds of sarcopenia development (OR = 1.70, 95%CI 1.0–2.9) [30]. It has been reported that WWI was positively associated with abdominal obesity, including total abdominal fat area, subcutaneous fat area, and visceral fat area [31]. Moreover, the negative correlation between WWI and muscle mass has been proven [14, 31]. WWI as a novel anthropometric indicator has the capability to reveal the association between obesity and UI. Therefore, it is reasonable to assume that an elevated WWI was linked to a greater likelihood of UI.

There are various anthropometric indexes assessing adiposity levels and BMI is the most widely used obesity parameter. However, it has been shown that BMI does not have the power to differentiate between lean mass and body fat percentage [32]. In addition, BMI cannot evaluate the locations of body fat deposition, leading to the inability of assessing abdominal obesity [33]. Moreover, BMI has a poor sensitivity for detecting obesity when BMI is no less than 30 kg/m^2^ [34]. Furthermore, the concept of the obesity paradox reveals that a higher BMI is correlated with a lower risk of cardiovascular events and better survival in patients with coronary artery disease [35]. Therefore, the limitations of BMI cannot be ignored while using it to evaluate obesity. WC as a simple indicator is used to evaluate central obesity. A cross-sectional study conducted for older women in southern Brazil demonstrated that compared to the lowest WC group (WC ≤ 79 cm), the odds of UI had a greater increase in the 79–86 cm group (OR: 1.98, 95%CI 1.13–1.45), the 86–94 cm group (OR: 2.07, 95%CI 1.16–3.69), and the highest WC group (WC > 94 cm) (OR: 2.24, 95%CI 1.26–3.99), revealing that WC was considered a significant obesity indicator for UI [36]. A cross-sectional survey in Korea showed that WC may be a more sensitive predictor for the relationship between obesity and UI in the elderly than BMI [37]. However, the obesity paradox was also observed when WC was used to explore the association between obesity and the clinical outcomes of heart failure [38]. These limitations of regular obesity indicators suggested that a more clinically applicable index was currently strongly needed. WWI proposed by standardizing WC based on body weight was positively correlated with cardiometabolic morbidity and mortality [13], indicating the phenomenon ‘obesity paradox’ was not obvious when WWI was utilized to assess obesity. Additionally, WWI is calculated with a simple formula, resulting in a convenient application in clinical examination. Moreover, in our study, the significantly stronger prediction for three types of UI was identified with WWI than BMI and WC. Therefore, WWI as a novel anthropometric index evaluating central obesity is greatly positively related to the odds of UI and has good predictive power for UI. More studies are needed to confirm whether WWI has a stronger prediction for the likelihood of other diseases than other traditional obesity indicators.

Our study used a large sample of data obtained from the NHANES database and took the sampling design and weighting into consideration for representing the general population in the United State. However, there are some limitations in the current study. First, due to the cross-sectional nature of this study, the causal association between WWI and UI cannot be explored. In addition, the NHANES database only represents the population in the U.S. and the association between WWI and UI is needed to be verified in different national populations by more investigations. Moreover, due to the limitation of questionnaire design for UI within the NHANES database, participants self-reported symptoms and history related to the three types of UI during interviews. This methodology likely resulted in an underestimation of the actual number of UI among individuals. Due to differences in participants' interpretation of questions, variations in educational background, and other potential factors, the design of self-reported questionnaire may have an impact on differences in survey individuals’ subjective assessment of their health statuses, thereby potentially introducing bias into the data analysis process. The utilization of a binary response format questionnaire within the database to evaluate patients with UI may increase bias because of subjective factors and overlook the variations in the severity of UI across the population. The binary and self-reporting assessment in this database warrants significant attentions. Lastly, although we adjusted several potential covariates in the current study, we could not entirely eliminate the impact of other conceivable confounding factors.

## Conclusion

To our knowledge, this is the first cross-sectional study exploring the relationship between WWI and UI in the United State adult population. In the current study, an elevated WWI was associated with a higher likelihood of UI. In addition, WWI had a stronger predictive power for UI compared to BMI and WC, indicating that WWI may be a better anthropometric index to evaluate UI. However, our findings are needed to be investigated by more prospective studies.

## Data Availability

Publicly available datasets were analyzed in the present study. All detailed data can be found here: www.cdc.gov/nchs/nhanes/.
